# Exploration of the anticancer properties of Caffeic Acid in malignant mesothelioma cells

**DOI:** 10.1007/s12032-025-02802-5

**Published:** 2025-06-10

**Authors:** Dayk Muratoglu, Gulseren Turhal, Busra Demirkan, Izel Nermin Baslilar, Nimet Sule Yuncu, Asuman Demiroglu-Zergeroglu

**Affiliations:** Department of Molecular Biology and Genetics, Faculty of Science, Gebze Technical University, 41400 Kocaeli, Turkey

**Keywords:** Caffeic acid, Malignant pleural mesothelioma, Cell proliferation, Apoptosis

## Abstract

Malignant Pleural Mesothelioma (MPM) is the most prevalent type of Mesothelioma and currently has no effective treatment options. This underscores the urgent need to explore new therapeutic agents and innovative strategies. Phenolic acids are significant natural compounds recognised for their effectiveness in treating various diseases, including cancer. This study evaluates the anti-carcinogenic properties of Cinnamic acid (CINN) and its derivative Caffeic acid (CA) in both MPM and non-cancerous mesothelial cells. Results show that CA exhibited greater efficiency than CINN in reducing cancer cell survival. This enhanced efficacy is primarily attributed to CA’s higher selectivity index and its ability to inhibit proliferation at lower concentrations. Consequently, further analysis was conducted using CA. The subsequent findings revealed that CA suppressed proliferative markers, Ki67 and PCNA, inhibited colony formation and wound healing in MM cells. Experiments also exposed that it suppresses the phosphorylation of ERK1/2 and AKT proteins in a concentration-dependent manner, while the phosphorylation of STAT3 remains unaffected. The pattern of protein phosphorylation and expression suppression by CA in 3D cells resembles that in 2D cells, although it occurred at higher concentrations. Additionally, CA significantly enhanced the expression of p53-regulated proteins p21 and p27, resulting in G2/M arrest in both SPC111 and SPC212 cell lines. Moreover, elevated concentrations of CA were associated with an increased number of dead cells, as demonstrated by DAPI/PI and AO/EtBr fluorescence staining. The increased Bax/Bcl-2 protein ratio, and BH3-only proteins (Bik and PUMA) and the cleavage of caspase-3 indicated that CA induces mitochondrial apoptosis. Our research with MM cells and three-dimensional micro-tumours suggests that CA may be a promising alternative for future MM therapies. However, it is vital to conduct high-throughput in vivo studies to elucidate further the potential importance of CA in treating this devastating disease.

## Introduction

CINN and CA are common components in various plant-based foods, such as vegetables and whole grains [1]. Anti-carcinogenic effect of these natural molecules has been demonstrated in some tumours [2, 3]. CINN reduces the viability of cells by causing an accumulation of melanoma cells in the S phase [4] and inhibits the growth of stem cell-like colon cancer cells [5]. Additionally, its derivative CA decreased the viability of breast cancer [6] and human melanoma cells [7]. CA-induced apoptosis was indicated in colorectal carcinoma and fibrosarcoma cell lines [8, 9]. Moreover, the suppression of angiogenesis and the downregulation of the PI3K/AKT and MAPK/ERK pathways were reported in CA-exposed hepatocarcinoma cells [10].

Malignant Mesothelioma, a highly aggressive and fatal tumour, originates from the thin layer of tissues covering the chest, abdominal cavity, and internal organs [11]. This disease is categorised into peritoneal, pericardial, and the most common type, Pleural Mesothelioma [12]. Approximately 90% of MPM cases are linked to prolonged exposure to carcinogenic mineral fibres, especially asbestos and erionite [13]. Corresponding studies have revealed that the uncontrolled signalling of KRAS, MAPK, PI3K/AKT/mTOR, and STAT3 pathways contributes to the carcinogenesis of MPMs in both mice and humans [14–17]. These results emphasise the importance of targeting these pathways in treating MPMs.

The standard systemic treatment for MPM currently involves a combination of Cisplatin with either Pemetrexed or Raltitrexed [18]. However, due to the limitations of existing treatments, there is a significant need to explore more effective and less toxic therapies [19, 20]. This research evaluates the anti-carcinogenic properties of the natural phenolic compound CA on MPM cells for the first time, assessing its potential as a future treatment option.

## Materials and methods

### Cell culture and preparation of substances

MeT-5A (CRL-9444) cell line was obtained from ATCC; SPC111 (11120716) and SPC212 (11120717) cells were purchased from ECACC. MeT-5A cells were grown in M199 medium (M5017, Sigma Aldrich) and supplemented with 10% FBS, (10270106, Gibco), 1% penicillin–streptomycin (15,140,122, Gibco), 1.24 g/L sodium bicarbonate (S8875, Sigma Aldrich), 0.4% L-Glutamine (G7513, Sigma Aldrich), 400 nM hydrocortisone (H0888, Sigma Aldrich), 20 mM HEPES (H0891, Sigma Aldrich), 3.3 nM EGF (E9644, Sigma Aldrich), and 870 nM insulin (I6634, Sigma Aldrich). Cancer cells were grown in DMEM: F12 medium (D8900, Sigma Aldrich) and supplemented with 10% FBS, 1% Penicillin–Streptomycin, and 1.24 g/L sodium bicarbonate. All cells were grown at 37 °C in a humidified atmosphere of 5% CO_2_. Master stocks of phenolic were prepared by dissolving powdered CINN (800235, Sigma Aldrich) and CA (C0625, Sigma Aldrich) in ethanol (070616185001, TEKKIM).

### Cell viability assay

Cells were seeded in 96-well plates (5 × 10^3^ cells/well) and incubated for 24 h, and then treated with different concentrations of CINN (0–5000 µM) and CA (0–3000 µM) for 72 h. After treatments, 3-(4,5-dimethylthiazol-2-yl)−2,5-diphenyltetrazolium bromide (MTT; M2128, Sigma Aldrich) solution was added to the wells to incubate for 2–3 h, at 37 °C. Then, the MTT solution was removed, and DMSO (1167431000, MERCK) was added to each well for 10 min. Colorimetric measurements were made at 570 nm using Varioskan Flash (Thermo-Fisher Scientific). The selectivity index (SI) was evaluated to assess the toxicity of the studied compounds on normal cells and to estimate their potential therapeutic benefits, which was calculated using the formula: SI = Composite IC_50_ in normal cell line/Composite IC_50_ in cancer cell line [21].

### Colony formation assay

SPC111 and SPC212 cells were seeded into 6‐well plates as 1 × 10^3^ cells/well overnight and then treated with a medium containing CA at different concentrations (0–100 μM) for 24 h. Afterwards, the media in each well was refreshed every three days. A minimum population of 50 or more cells was considered as a colony. Finally, at the end of the twelve days, the colonies were fixed with 4% formaldehyde and stained with 0.1% crystal violet (in dH_2_O). Next, the wells were rinsed with dH_2_O to remove residues and formed colonies were counted. The plating efficiency values were calculated with the formula: *Plating Efficiency* = *(Number of colonies formed/Number of cells plated)* × *100.*

### Wound healing assay

Cell lines were plated in 96 wells (2,5 × 10^4^ cells/well) for 24 h. Next, the medium was removed, a straight line was scratched across the well with the tip of a pipette to establish the wound area and the wells were washed with 1X PBS. After that, the medium containing 1% FBS and CA (25, 50, 100 µM) was added to the wells, and then, the wound area was visualised in 48 h with an inverted microscope (Leica DM IL LED) and the LAS V4.6 program. The images obtained for each sample were quantitatively analysed with ImageJ and the “MRI Wound Healing Tool. ijm” plugin of this software, and the cell-free area in the acquired images was calculated, as previously mentioned.

### Construction of 3D spheroids

3D-MPM spheroids were generated using the hanging drops technique. SPC111 and SPC212 cells were plated in ultra-low attachment 96-well plates as 1 × 10^4^ cells/well and grew at 37 °C for 5–6 days to reach a spheroid structure with a 350–400 μm diameter. Then, different concentrations of CA were applied to cells for 24 h and calculated using the ImageJ program.

### Flow cytometry analysis

#### Ki67 and PCNA

Cell lines were seeded in 6-well plates (2 × 10^5^ cells/well) and incubated O/N. Subsequently, the medium was substituted with a fresh medium comprising CA in concentrations of 50 and 100 µM, which was then left to incubate for 24 h. Following the incubation process, the cells were collected using trypsin and subjected to centrifugation at room temperature for 5 min. Thereafter, 70% ice-cold ethanol was added to the cell pellets in a dropwise manner, and the samples were then incubated at a temperature of −20 °C for a minimum 2 h. Fixed cells can be stored at −20 °C up to 60 days. Following the washing of fixed cells with wash buffer (1% FBS, 0.09% NaN3 in PBS, pH 7.2) on two occasions, the cells were centrifuged at 1000 rpm for 10 min. The supernatant was then discarded. The cells were resuspended in 100 µl of wash buffer, after which the diluted Ki67 and PCNA antibodies were added. The samples were then subjected to an incubation period of 30 min at room temperature. Following the incubation process, the samples were analysed with BD-Accuri C6 and its software (BD Bioscience).

#### Cell cycle profile

Cell lines were incubated in 6-well plates (2 × 10^5^ cells/well) for O/N, and then the medium was replaced with a serum-free medium to synchronise the cells. Next, cells were incubated with a complete medium containing CA (25, 50, 100 µM) for 24 h and were transferred to tubes for centrifugation. The pellet was then gently washed with 1 × PBS and dissolved with chilled 70% ethanol. All samples were fixed at + 4 °C for 30 min and stored at −20 °C. To remove ethanol, samples were centrifuged at 1000 g at + 4 °C for 5 min and then dissolved in 1X PBS. The working solution was prepared by adding RNase (10,109,134,001, Roche) to the master stock prepared with PI and 10% Triton X-100 (T8787, Sigma Aldrich) in 1 × PBS. The samples incubated at 37 °C for 30 min in the dark were centrifuged to remove the dye and then dissolved in 1 × PBS at RT. All measurements and calculations were achieved with BD-Accuri C6 and its software (BD Bioscience), respectively.

### Dual fluorescent staining

Cells were seeded in 96-well plates (1 × 10^4^ cells/well) for O/N and then treated with different concentrations of CA (25, 50, 100 µM) for 24 h. Two different fluorescent staining techniques (AO/EtBr and DAPI/PI) were used to morphologically and quantitatively distinguish viable and dead cells. Imaging was performed with a magnification of 20 × using proper fluorescent channels in the ZOE Fluorescent Cell Imager (Bio-Rad). The morphology and the number of cells were analysed by ImageJ. For *DAPI/PI,* PI was diluted in 1 × PBS with a final concentration of 25 μg/ml, added to the wells for 10 min and then washed with 1 × PBS before 4% formaldehyde (7041, JT Baker) fixation in the dark. DAPI was prepared according to the manufacturer's protocol. 1 µg/ml was added to the wells for the final concentration and then washed with 1 × PBS. The blue and red fluorescent channels were used to analyse the cell morphology. *AO/EtBr dye* (235,474/E7637, Sigma Aldrich) prepared in 1 × PBS at a 1:1 ratio was added to each well in the medium and was removed after 15 s before imaging was performed with green and red fluorescent channels.

### Western blot analysis

1.2 × 10^6^ cells were seeded for O/N and then exposed to 25, 50, or 100 µM of CA for 24 h. Next, the samples were harvested with freshly prepared lysis buffer containing 150 mM NaCl, one mM MgCl_2_ (M4880, Sigma Aldrich), 10% glycerol (G5516, Sigma Aldrich), 20 mM Tris Base (pH:8), (T1503, Sigma Aldrich), 1% Triton X-100 (T8787, Sigma Aldrich), 10% SDS (194831, MP), and 1% protease-phosphatase inhibitor (1862495, Thermo-Fisher Scientific. The protein concentrations were determined using the BCA Protein Assay Kit (23227, Thermo-Fisher Scientific) and Varioskan Flash (Thermo-Fisher Scientific). Next, the total protein content was separated by 10–12% SDS-PAGE and transferred to a PVDF membrane, which was blocked with 5% skim milk powder (A0830, AppliChem) in TBS-T obtained by adding 0.1% Tween 20 (A4979, AppliChem) to 1X TBS and incubated with primary antibodies of Cell Signalling Technology: p-ERK1/2 (#8544), t-ERK1/2 (#9107), p-AKT (#4060), t-AKT (#4691), p-STAT3 (#9145), t-STAT3 (#12,640), p21 (#2947), p27 (#3686), p-p53 (#9286), t-p53 (#9282), Bax (#5023), Bcl-2 (#15,071), cleaved caspase-3 (#9664), Bik (#4592), Puma (#4976). The anti-rabbit (#7074) and anti-mouse (#7076) were used as secondary antibodies. The Cyclophilin B (PA1-027A, Thermo-Fisher Scientific) was a loading control antibody. ECL Plus Western Blotting Substrate kit (32132, Thermo-Fisher Scientific) and ChemiDoc XRS + (Bio-Rad) system were used for detection, and the density of protein bands was analysed with Image Lab 6.0 Software (Bio-Rad).

### Statistical analysis

Each experiment was conducted thrice with multiple replicated samples, and then GraphPad Prism 8.3.0 was used for all statistical and graphical analyses.

## Results

### The viability of MPM cells diminished in a concentration-dependent manner after treatment with CINN and CA

**Fig. 1:**
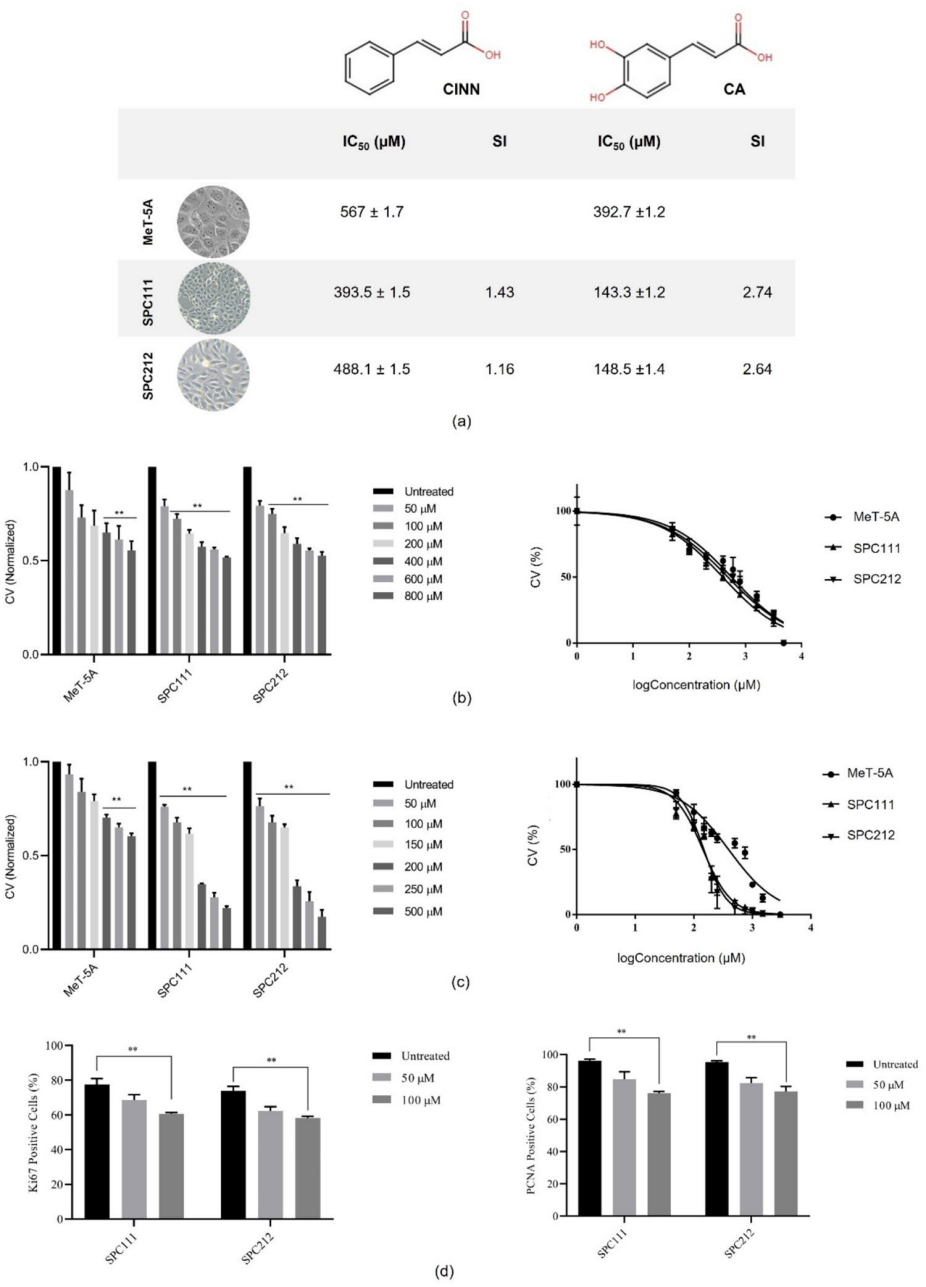
The cell viability after treatments with CINN and CA. MeT-5A, SPC111, and SPC212 cells were exposed to different concentrations of CINN and CA for 72 h, and the cell viability (CV) was measured using the MTT assay. IC_50_ and SI values of cell lines (**a**), viability graphs represent the effect of CINN (**b**) and CA (**c**) in all three cell lines. Ki67 and PCNA expression levels were analysed with flow cytometry (**d**) (*n* = 3, **p < 0.01)

To investigate the impact of CINN and CA on the viability of MPM and non-cancerous MeT-5A cell lines, an MTT assay was utilised for 72 h. The IC_50_ values were calculated, as it is shown in Fig. 1. The selectivity index (SI) was calculated as *1.43* and *1.16* for CINN and *2.74* and *2.64* for CA in SPC111 and SPC212 cells, respectively*.* The number of non-cancerous mesothelial cells decreased when treated with CA from approximately 400 µM. However, this was observed in cancer cells starting from a concentration of 100 µM (**p < 0.01). These results indicated that CA is more cytotoxic to MPM cells than MeT-5A cells and more effective on MPM cells than CINN. Therefore, based on IC_50_ and SI values, succeeding analyses were continued only with CA in later stages.

Ki-67 and Proliferating Cell Nuclear Antigen (PCNA) are well-established markers that are commonly employed to assess cellular proliferation. Ki-67, present during all active phases of the cell cycle (G_1_, S, G_2_, and M) but absent in resting (G_0_) cells, serves as an indicator of actively cycling populations. Similarly, PCNA is a processivity factor for DNA polymerase δ, which is essential during DNA replication in the S phase. Furthermore, the expression levels of PCNA are also tightly linked to cell proliferation. Here, the observed reduction in MPM cellular proliferation following treatment with CA was further substantiated by a notable decrease in both Ki-67 and PCNA levels, indicating a direct impact on the cell cycle progression and the proportion of actively dividing cells.

### CA inhibited the formation of colonies and delayed wound healing in MPM cells

**Fig. 2:**
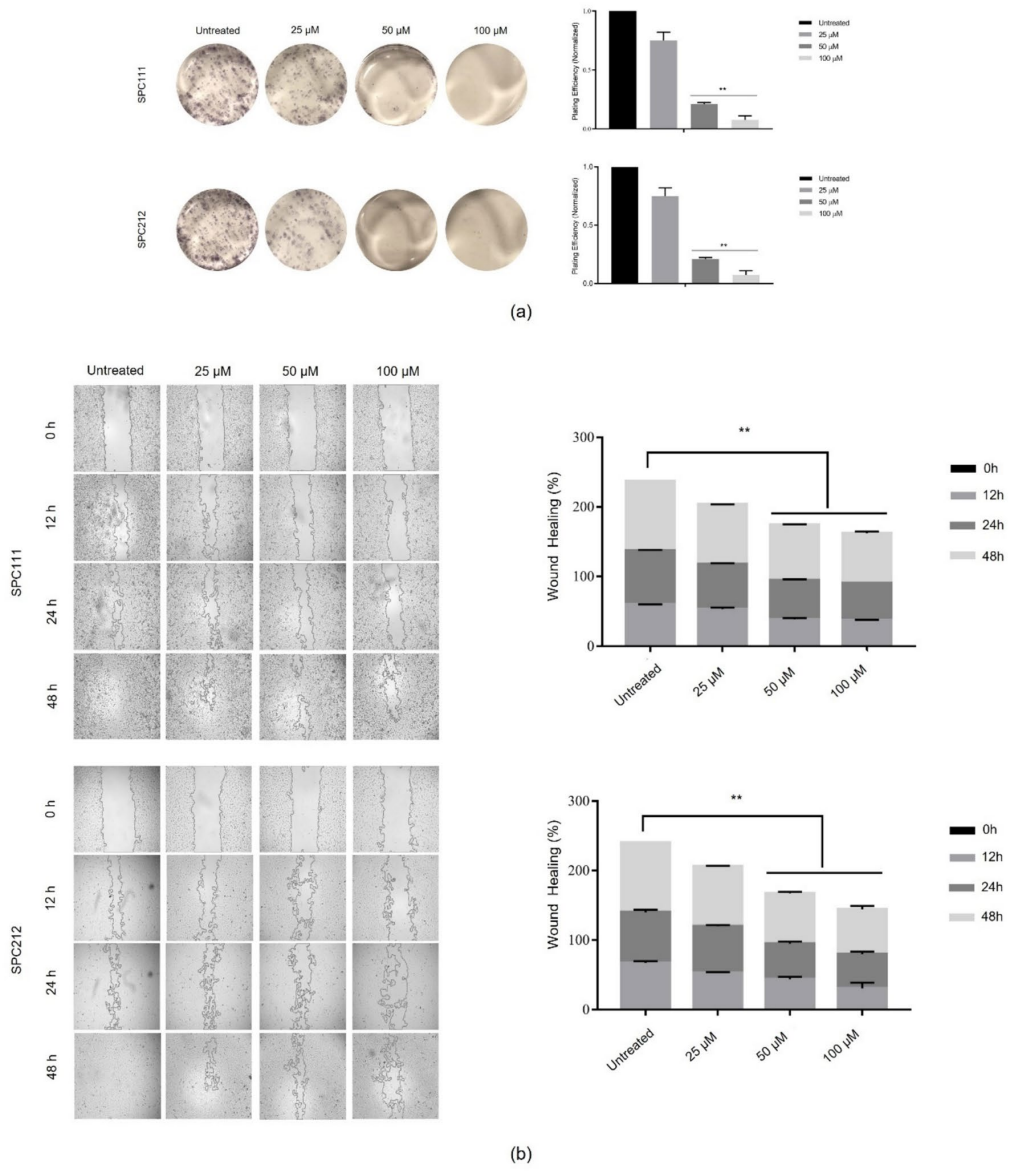
Formation of SPC111 and SPC212 colonies and delay of wound healing. Both MPM cell lines were exposed to 0, 25, 50, and 100 µM concentrations of CA. The reduction of the colonies was observed at 5 × magnification under the microscope (**a**). Cellular wound closure was evaluated in SPC111 and SPC212 cells between 0 and 48 h (**b**). Formed colonies and healed wound areas were calculated using the ImageJ program (*n* = 3, **p < 0.01)

To evaluate the effect of CA on colony formation and wound healing, cancer cells were treated with CA at concentrations ranging from 0 to 100 µM. The number of colonies decreased with increased concentration of CA, and a significant effect was observed at 50 µM in SPC111 and SPC212 cells, as shown in Fig. 2a. Likewise, the wound closure delay was observed at the same concentration and above in both cell lines. Besides, compared to untreated ones, the CA-treated cells healed over 48 h (Fig. 2b). These findings suggest that wound healing delay in MPM cells through CA was time- and concentration-dependent.

### CA led to a decrease in spheroid size and suppressed growth, survival, and proliferation signals in both 2D and 3D cultures

**Fig. 3:**
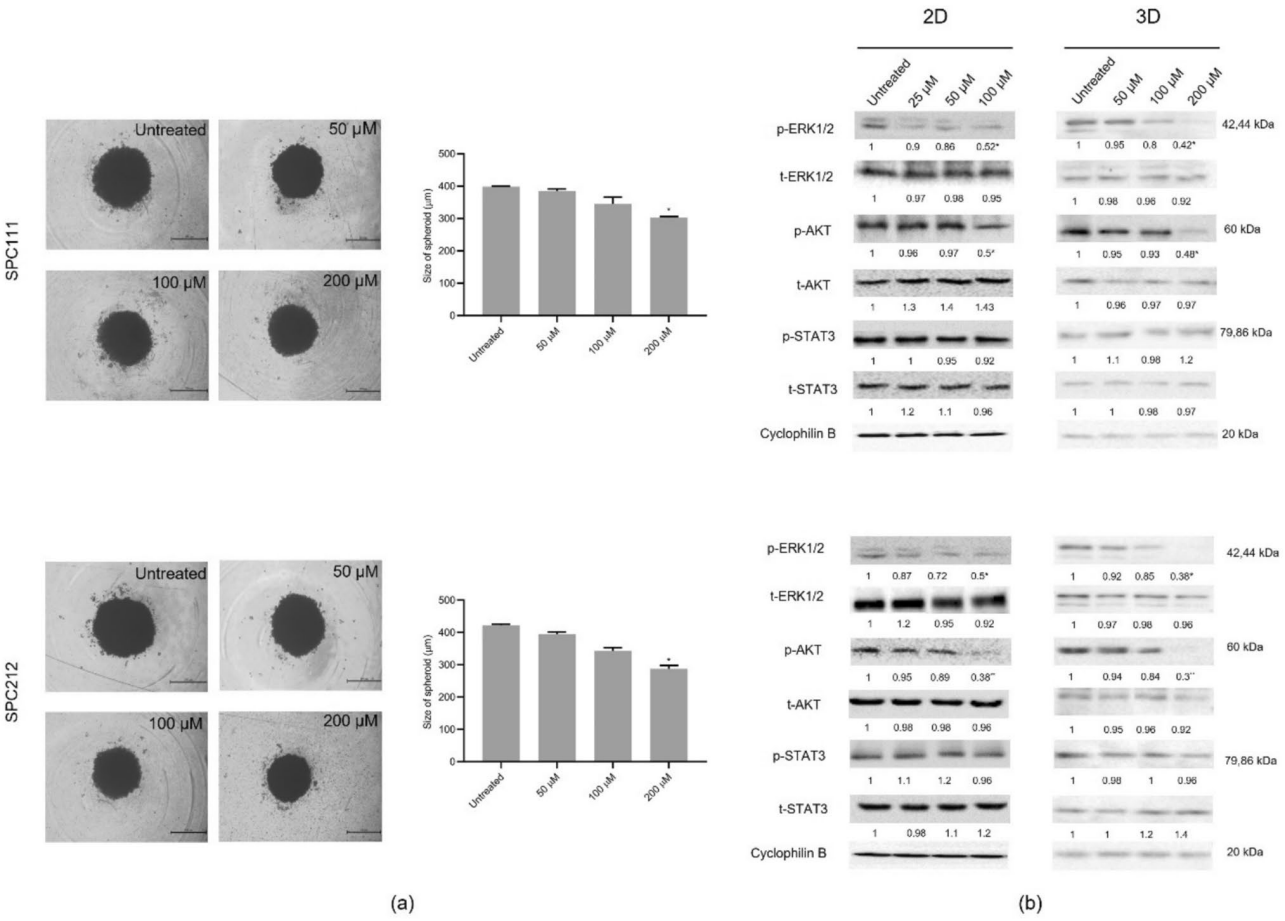
The Effect of CA on the spheroid size and the signal transduction proteins involved in cell growth/survival and proliferation*.* SPC111 and SPC212 cells were exposed to CA for 24 h and then analysed under the microscope with the LAS v4 program (5 × magnification) (**a**). After treatments, the phosphorylation and expression profiles of ERK1/2, AKT, and STAT proteins were detected by western blot analysis (**b**) (*n* = 3, *p < 0.05, **p < 0.01)

To assess the effect of CA on the sizes of 3D micro-tumours, sequential concentrations (0–200 µM) were applied for 24 h, and then the size of spheroids was analysed with the Image J program. As shown in Fig. 3a*,* significant shrinkage was observed in 3D cells treated with 200 µM CA concentrations. Subsequently, upon comparing CA's ability to affect phosphorylation and expression of proteins in both dimensions, not the expression but significant suppression of ERK1/2 and AKT phosphorylation were observed in monolayer cells with the application of 100 µM. However, this level of suppression only occurred in spheroid cells when CA was applied at twice the concentration, as represented in Fig. 3b (*p < 0.05, **p < 0.01). Interestingly, CA treatments did not affect phosphorylation and expression levels of STAT3 protein in SPC111 and SPC212 in either 2D or 3D cells. However, when MPM cells were exposed to mM concentrations of CA, the phosphorylation of STAT proteins could be inhibited (data not shown here).

### CA-induced G2/M phase arrest, associated with increased levels of p21 and p27, is regulated by p53 activation

**Fig. 4:**
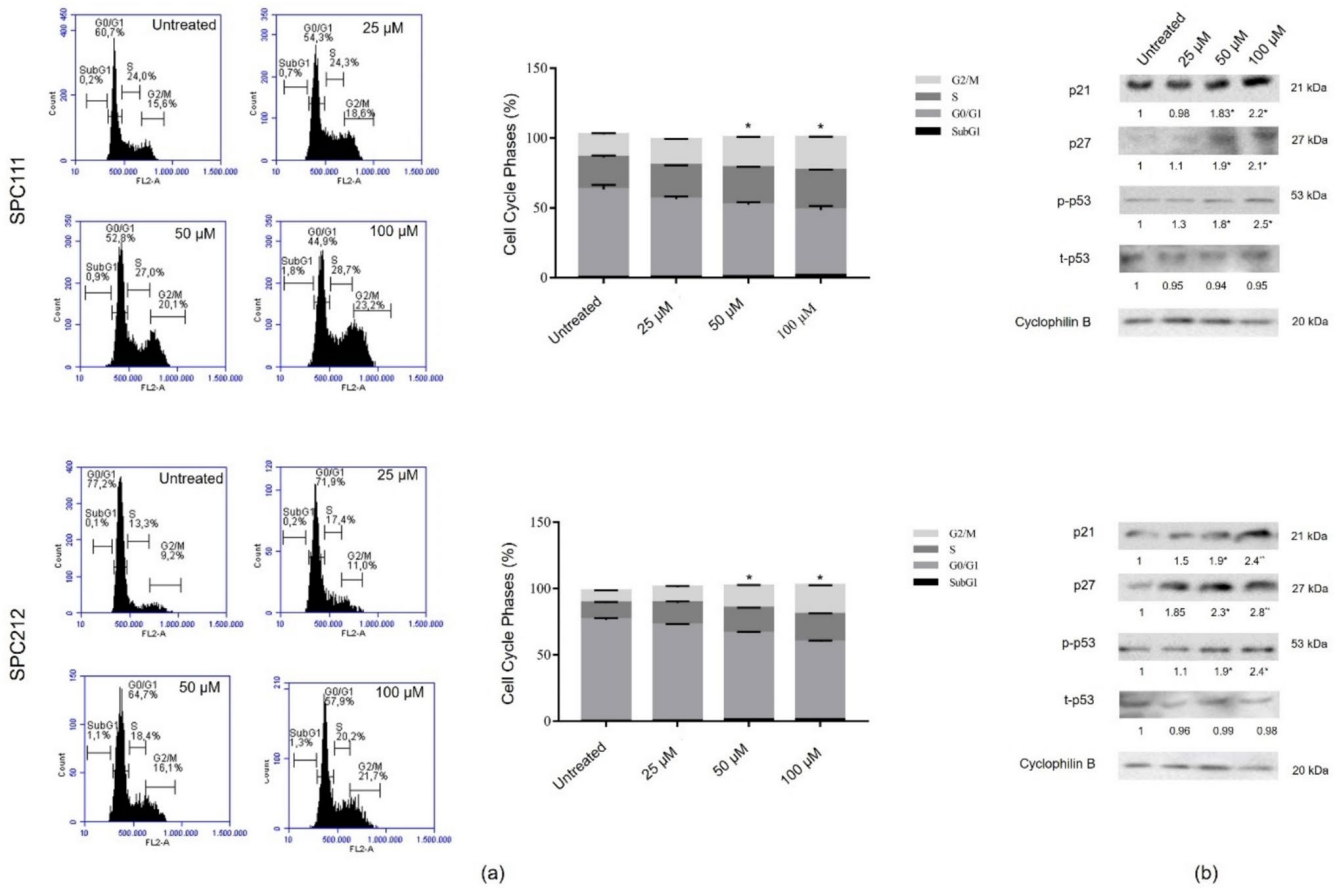
Alteration of the cell cycle profile of MPM cells. The concentration-dependent variation of the cell cycle profile of SPC111 and SPC212 cells was examined by PI staining with flow cytometry in 24 h (**a**). The western blot analysis of checkpoint proteins p53, p21, and p27 after cells were exposed to the consecutive concentrations of CA (**b**) (*n* = 3, *p < 0.05, **p < 0.01)

To determine the influence of CA on the cell cycle profile, cancer cells were treated with a concentration range of 0–100 µM and analysed by PI staining accompanied by flow cytometry. As shown in Fig. 4a*,* both cancer cells were arrested at the G2/M phase of the cycle compared to untreated control cells. The accumulation of cells at G2/M was significantly increased from 50 µM concentrations in 24 h. Moreover, phosphorylation and/or expression of p53 and two cyclin-dependent kinase inhibitor proteins, p21 and p27, were also evaluated in SPC111 and SPC212 cells. Our findings reveal a substantial increase in p21 and p27 expression within MPM cells following exposure to 50 µM or more of CA (Fig. 4b). Additionally, phosphorylation of p53 increased, although no significant change was detected in total protein levels (*p < 0,05 and **p < 0,01).

### Mitochondrial apoptosis was triggered in MPM cells after treatment with CA

**Fig. 5:**
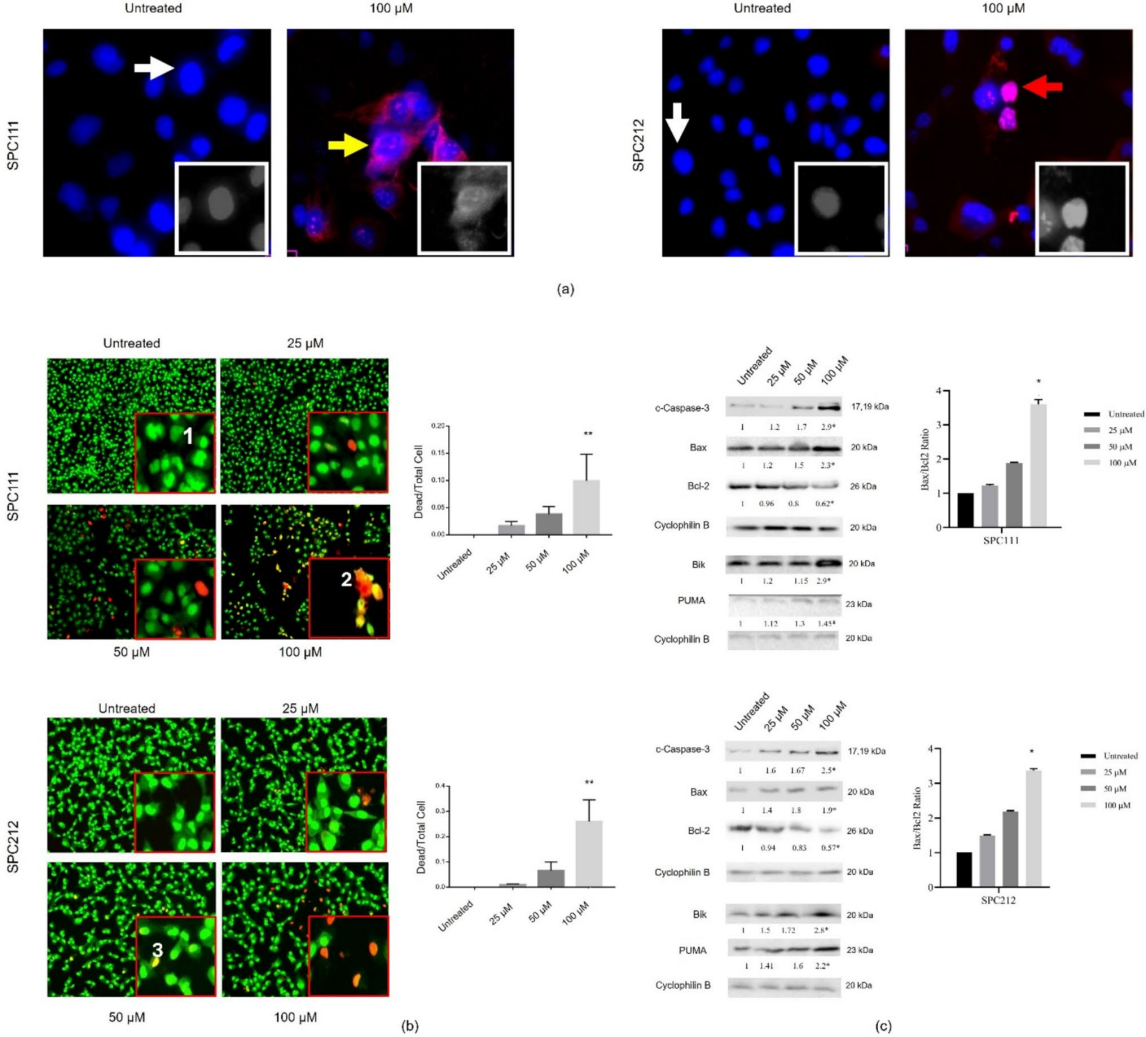
Apoptotic effect of CA on SPC111 and SPC212 cells*.* Cell death morphology was determined by DAPI/PI (**a**) and AO/EtBr staining (**b**). In DAPI/PI-stained samples, the intact nucleus is shown by a white arrow; chromatin condensation and nuclear condensation are displayed by the yellow and red arrows, respectively. In AO/EtBr staining, the nuclei of viable and non-apoptotic cells were stained green (1); Apoptotic cell morphology manifests as yellow and orange dots with fragmented nuclei and condensed chromatin structures (2), unlike the orange/red-stained necrotic cells (3). The cellular protein levels were demonstrated in a range of 0–100 µM concentrations, and the Bax/Bcl-2 ratio (**c**) was calculated according to the expression level of the proteins. In addition, BH3-only proteins Bik and PUMA expressions were increased in CA-treated cells (*n* = 3, *p < 0.05) (20 × magnification; scale bar, 25 µm)

Next, the impact of CA on cell death was assessed in SPC111 and SPC212 cells within the concentration range of 0–100 µM. The morphological examination was initially conducted using DAPI/PI staining, followed by morphological and quantitative analysis of AO/EtBr. These results indicated that the number of viable cells decreased, and dead cells increased in both cancer cells treated with CA. The resulting images from DAPI/PI showed that apoptotic and necrotic cells were present following treatment with 100 µM of CA (Fig. 5a). Then, AO/EtBr staining was carried out with several concentrations of CA and the apoptotic morphology was observed when the concentration of CA reached 50 µM in 24 h, and a significant increase in the number of death cells was detected at 100 µM (Fig. 5b). Both staining results indicated that CA induces apoptosis in SPC111 and SPC212 cells in a concentration-dependent manner. Moreover, the reduction in Bcl-2 protein expression coupled with a concentration-dependent elevation in Bax protein expression strongly suggests the stimulation of mitochondrial apoptosis. Furthermore, the presence of activated Caspase-3 in both cell types upon exposure to 100 μM CA confirmed the process. Figure 5c demonstrates the Bax/Bcl-2 ratio, indicating increased sensitivity to apoptosis, which was calculated based on the ratio of protein expression levels.

## Discussion

Phenolic acids and their derivatives have recently attracted interest in drug research due to their natural origins and biological and pharmacological benefits. This study examined the anticancer effects of two phenolic acids, CINN and its derivative, CA, in MPM cells and mesothelial cells. A significant concentration-dependent reduction was detected in the viability of cancer cells. However, this effect was only observed when higher concentrations of phenolic compounds were applied to non-cancerous counterpart cells. In other words, CINN and CA exhibited selective cytotoxicity against SPC111 and SPC212 cells when compared to non-tumour MeT-5A cells. While both compounds demonstrated anti-proliferative effects on cancer cells, further investigation was continued solely with CA due to its effectiveness at lower concentrations and its higher selectivity index value for MPM cells.

The levels of Ki-67, a common marker of cellular proliferation [22], and PCNA, a component of the DNA replication and repair machinery [23] were examined. In addition to Ki-67, it is well known that PCNA expression serves as a marker of cell proliferation because cells remain in the G1/S phase for long periods while proliferating. As shown in the Results section, Ki-67 and PCNA levels were decreased in CA-treated cells compared to their untreated counterparts, which is consistent with our previous results.

It is widely recognised that uncontrolled activation of signalling molecules contributes to carcinogenesis. Therefore, many potential drugs aim to block aberrant proliferative and survival signalling pathways, such as ERK1/2, PI3K/AKT, and STAT3 [24]. It has been previously reported that CA suppresses ERK phosphorylation and downstream signalling molecules, including RSK2, Elk1, and c-Myc, induced by UV irradiation in primary mouse and human keratinocyte cells [25]. Others have shown that this phenolic compound can mimic the effects of anti-oestrogens by altering key growth regulatory signals involving ER/cyclin D1 and IGF1R/p-AKT, thereby reducing the proliferation of breast cancer cells [26]. Moreover, the co-administration of CA and AKT activator effectively reduced the inhibitory effects of CA on the formation of colorectal cancer stem cell spheres and AKT signalling [27]. Similarly, treatment with CA reduced the phosphorylation of ERK1/2 and AKT in MPM cells.

Higher concentrations of CA were required to inhibit phosphorylation in 3D-MPM spheroids compared to monolayers; however, similar patterns of inhibition were observed. Current research indicated that tumour cells exhibit reduced sensitivity to drugs in 3D cultures compared to 2D cultures. This reduced sensitivity may be attributed to factors such as limited access to compounds in the medium, hypoxic conditions, and alterations in the cell cycle [28]. Moreover, it has been highlighted that in vitro conditions and culturing methods play a significant role in influencing cellular metabolic activity, proliferation, and drug sensitivity [29]. Nevertheless, it is worth noting that 3D cell cultures, which maintain cell densities similar to those found in native tissues, can produce drug responses that closely resemble those observed in solid tumours [28].

Some studies have shown that CA suppressed hypoxia-induced STAT3 phosphorylation (in Y705), nuclear translocation of STAT3, HIF1α induction, and VEGF expression in human renal cancer cells [30]. Additionally, after CA treatment, STAT3 phosphorylation levels decreased in macrophage cells [31]. This modulation of the JAK/STAT3 pathway by CA treatment activated the apoptosis mechanism in a UVB-induced skin cancer model in mice [32]. After exposure to CA, neither the phosphorylation nor the overall expression of STAT3 proteins changed in MPM cells. This suggests that the growth-inhibitory effect of this phenolic compound may be independent of the STAT3 pathway. However, when CA was applied at millimolar concentrations, it was found to suppress STAT3 phosphorylation in MPM cells (data not shown). These results indicate that the suppression of the STAT3 pathway by CA is dependent on both concentration and the specific context of the cell type.

It is reported that CA decreases cell migration in MDA-MB-231 and MCF-7 breast cancer cells, as well as in malignant human keratinocytes [33, 34]. The experiments on colony formation and wound healing demonstrate that CA inhibits the growth, proliferation, and cell migration of SPC111 and SPC212 cells. Previous studies have shown that reduced ERK1/2 signalling in MM is linked to decreased cellular invasion and migration [35]. CA may suppress the growth, proliferation, and migration of MPM cells in a concentration-dependent manner by inhibiting the phosphorylation of the ERK1/2 and AKT proteins.

The cytostatic and apoptotic effects of CA have been demonstrated in relevant studies. For instance, cell accumulation at the sub-G1 and G0/G1 phases was observed in NSCLC and pharyngeal cancer cells, respectively [36, 37]. CA treatment caused G2/M arrest in SPC111 and SPC212 tumour cells starting from 50 μM, along with a concentration-dependent increase in p21 and p27 protein expressions, which are known to inhibit kinase activity and cell cycle progression [38–40]. Furthermore, CA-stimulated apoptosis has been reported in K562 chronic myeloid leukaemia cells [41] and SK-Mel-28 melanoma cells [7]. The Bcl-2 family is known to play a critical role in both anti-apoptotic and pro-apoptotic processes [42]. Bax and PUMA both promote apoptosis and are direct targets of p53 [43]. Stress-activated Bax can counteract the anti-apoptotic effects of Bcl-2, and cells lacking Bax were resistant to p53-dependent apoptosis [44]. Our morphological analysis revealed that CA induced cell death in MPM cells. Additionally, we observed increased p53 phosphorylation, a high Bax/Bcl-2 ratio, and the cleavage of Caspase-3 after treatment. These findings demonstrate the occurrence of concentration-dependent mitochondrial apoptosis in both SPC111 and SPC212 cells.

This study examined the effects of CA on MPM cells, focusing on areas such as cell growth, proliferation, the cell cycle, cell death, and the key proteins involved in these biological processes. The results are promising, and the next step is to validate these findings through in vivo analyses.

## Data Availability

No datasets were generated or analysed during the current study.

## Abbreviations

MPM: Malignant pleural mesothelioma
CINN: Cinnamic acid
CA: Caffeic acid
ERK1/2: Extracellular regulated kinase1/2
AKT: Protein kinase B
STAT3: Signal transducer and activator of transcription 3
3D: Three dimension
DAPI/PI: 4′,6-Diamidino-2-phenylindole/Propidium iodide
AO/EtBr: Acridine orange/ethidium bromide
Bcl-2: B-cell lymphoma
Bax: Bcl-2 associated X protein
